# Betulinic Acid in Complex with a Gamma-Cyclodextrin Derivative Decreases Proliferation and *in Vivo* Tumor Development of Non-Metastatic and Metastatic B164A5 Cells

**DOI:** 10.3390/ijms15058235

**Published:** 2014-05-09

**Authors:** Codruta Soica, Corina Danciu, Germaine Savoiu-Balint, Florin Borcan, Rita Ambrus, Istvan Zupko, Florina Bojin, Dorina Coricovac, Sorina Ciurlea, Stefana Avram, Cristina Adriana Dehelean, Teodora Olariu, Petru Matusz

**Affiliations:** 1 Faculty of Pharmacy, Victor Babeş University of Medicine and Pharmacy, 2 EftimieMurgu, 300041 Timisoara, Romania; E-Mails: codrutasoica@umft.ro (C.S.); fborcan@yahoo.com (F.B.); dorina_gheorgheosu@yahoo.com (D.C.); sorinaciurlea@yahoo.com (S.C.); stefana.feflea@gmail.com (S.A.); cadehelean@umft.ro (C.A.D.); 2 Institute of Pharmaceutical Technology, University of Szeged, 6 Eotvos Ut., H-6720 Szeged, Hungary; E-Mail: rita-techno@freemail.hu; 3 Department of Pharmacodynamics and Biopharmacy, University of Szeged, 6 Eotvos Str., H-6720 Szeged, Hungary; E-Mail: zupko@pharm.u-szeged.hu; 4 Faculty of Medicine, Victor Babeş University of Medicine and Pharmacy, 2 EftimieMurgu, 300041 Timisoara, Romania; E-Mails: florinabojin@umft.ro (F.B.); matusz@umft.ro (P.M.); 5 Faculty of Medicine, Vasile Goldis University, 310045 Arad, Romania; E-Mail: olariu.teodora@yahoo.com

**Keywords:** betulinic acid, DSC, X-ray diffraction, SEM, MTT test, C57BL/6J mice

## Abstract

Betulinic acid, a very promising anti-melanoma agent, has very low water solubility that causes low bioavailability. To overcome this inconvenience, a highly water-soluble cyclodextrin was used (octakis-[6-deoxy-6-(2-sulfanyl ethanesulfonic acid)]-γ-cyclodextrin). The complex was physico-chemically analyzed using differential scanning calorimetry (DSC), X-ray and scanning electron microscopy (SEM) methods and then *in vitro* tested for its antiproliferative activity by the MTT assay and by cell cycle analysis. Finally, the complex was tested *in vivo* using an animal model of murine melanoma developed in C57BL/6J mice, where it caused a reduction in tumor volume and weight. The study revealed the beneficial influence of betulinic acid inclusion into the cyclodextrin in terms of antiproliferative activity and *in vivo* tumor development.

## 1. Introduction

**Figure 1.**
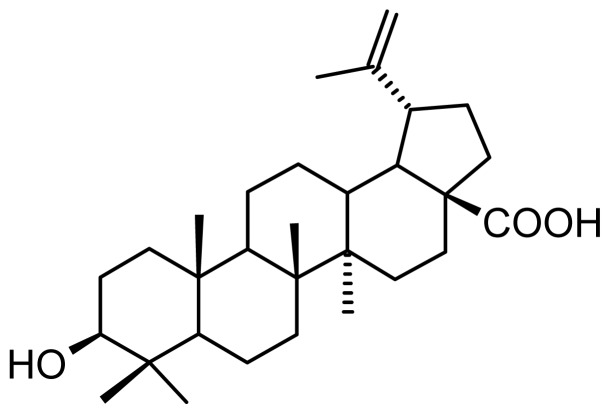
Betulinic acid (BA) molecular structure.

Betulinic acid (BA, Figure 1) was discovered as an antimelanoma agent in 1995 by Pisha *et al.* [1]. Since then, it has been proven as an antitumor agent on many other cell lines: glioma, medulloblastoma, glioblastoma, ovarian carcinoma, lung carcinoma, cervical carcinoma, head and neck carcinoma, hematological malignancies, and skin carcinoma [2,3]. The most important advantage of BA as an anticancer agent is its selective activity on tumor cells with no cytotoxic activity on normal cells [4]. Based on these facts, BA has been introduced in the RAID (Rapid Access to Intervention Development) program by the United States NCI (National Cancer Institute) [5]. One important facet in anticancer treatment is finding a way to prevent or stop metastasis development. BA has been proven to act more aggressively on human metastatic C8161 melanoma cells than their non-metastatic equivalent (C8161/neo 6.3) [6]. Additionally, in combination with vincristine, BA was able to prevent metastasis to the lungs in a B16F10 melanoma model in mice [7].

All research regarding the anticancer activities of BA and other pentacyclic triterpenes is hampered by low water solubility, which greatly limits therapeutic applications as well as bioavailability [2,8]. To overcome this solubility issue, cyclodextrin (CD) complexation has been accomplished using β- and γ-derivatives with high water solubility [9,10]. γ-Derivatives were described as the most appropriate host molecules for the bulky structure of BA (Figure 1) [11], and the complexes were physico-chemically characterized and *in vitro*/*in vivo* tested [9–12]. In spite of a significant increase in water solubility, which led to an optimized bioavailability, the stability constants of the final complexes were rather low [9–12]. Because of this result, an efficient targeted delivery of the active compound, essential in cancer therapy [13], could not be accomplished. Based on this information, semisynthetic γ-derivatives with hydrophobic substituents were introduced as host molecules and evaluated using NMR techniques [14]. An even better water solubility was achieved and, more importantly, a very strong complex was formed [14], capable of retaining the active substance long enough to reach its final delivery site.

The present paper aims to present a comprehensive physico-chemical and biological characterization of BA complexed with octakis-[6-deoxy-6-(2-sulfanyl ethanesulfonic acid)]-γ-CD (GCDG) using *in vitro* tests on tumor cell lines and *in vivo* tests on experimental animal models.

## 2. Results and Discussion

### 2.1. Scanning Electron Microscopy (SEM)

**Figure 2.**
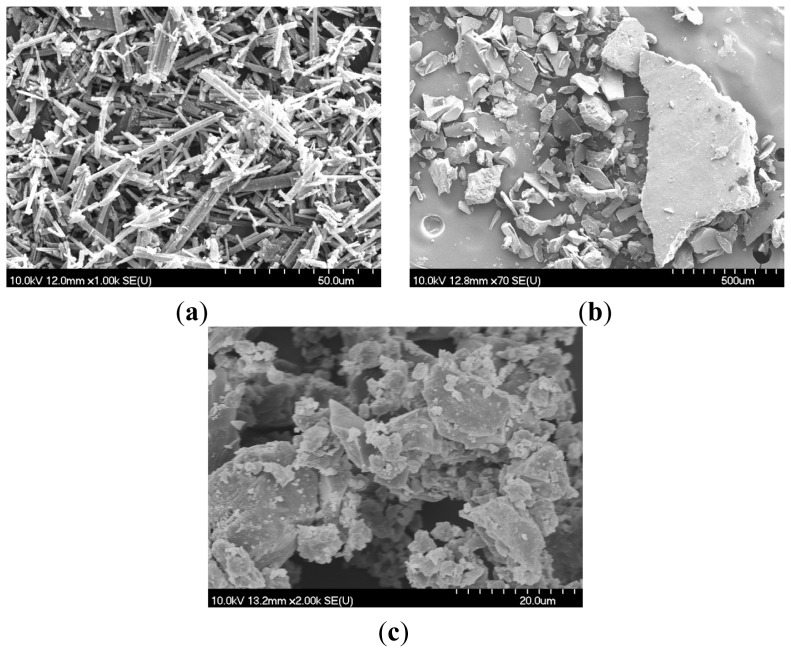
Scanning electron microscopy (SEM) pictures of (**a**) Betulinic acid (BA); (**b**) octakis-[6-deoxy-6-(2-sulfanyl ethanesulfonic acid)]-γ-cyclodextrin (GCDG); and (**c**) BA:GCDG, 1:1 complex.

Scanning electron microscopy (SEM) is successfully used in the evaluation of the microscopic aspects of the active compound, complexing cyclodextrin and complex formation (Figure 2a–c).

Although SEM is not a decisive method in terms of absolutely confirming complex formation, the method helps assess the presence of only one component in the final products. In Figure 2a, BA exhibits crystalline particles characterized by regular parallelepiped shape and similar size. The cyclodextrin (Figure 2b) shows amorphous particles of irregular shape and size, and some of them are highly aggregated. The physical mixture of BA and GCDG (not shown) contains both crystalline and amorphous particles adhering to mutual surfaces. A dramatic change in morphology and crystallinity degree is noticed in the 1:1 kneaded complex (Figure 2c), revealing a presumable interaction in the solid state. SEM pictures of the final product exhibit small particles with a clear tendency to aggregate, a specific behavior for amorphous particles of a single component, therefore leading to the conclusion that real complexing took place.

### 2.2. Differential Scanning Calorimetry (DSC)

**Figure 3.**
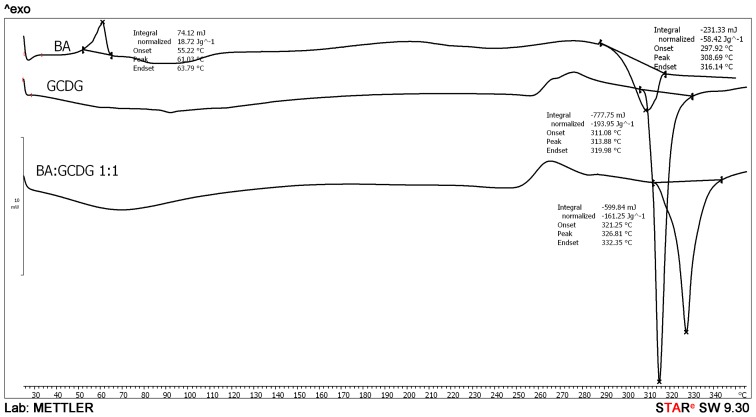
Differential scanning calorimetry (DSC) curves of BA, octakis-[6-deoxy-6- (2-sulfanyl ethanesulfonic acid)]-γ-cyclodextrin (GCDG) and their 1:1 complex. As carrier gas argon was used, the heating rate was maintained at 5 °C/min, while the sample weight was 2–5 mg. Experiments were conducted from 25 up to 300 °C.

Differential scanning calorimetry (DSC) analysis was performed in a temperature range of 0–350 °C (Figure 3).Within this range, BA revealed a very small exothermic peak at approximately 60 °C and a strong endothermic peak at approximately 306 °C, corresponding to the decomposition of the substance.

The cyclodextrin also presented a very sharp endothermic peak at approximately 313 °C, indicating a strong endothermic process accompanying its decomposition. In the physical mixture (not presented), the DSC curve is practically the sum of the individual curves of both compounds. In contrast, for the 1:1 kneaded product, the DSC curve exhibits a very smooth endothermic process before 100 °C, as determined by water evaporation; moreover, the complete disappearance of both BA peaks and the presence of the cyclodextrin peak are observed, with a shift of position from 313–326 °C. This new peak, presumably attributed to the complex decomposition, indicates the interaction that took place between the two components with the formation of the 1:1 complex. The disappearance of the BA peaks proves that the active compound is completely included in the cyclodextrin cavity and is unable to perform as the pure drug.

### 2.3. X-ray Analysis

**Figure 4.**
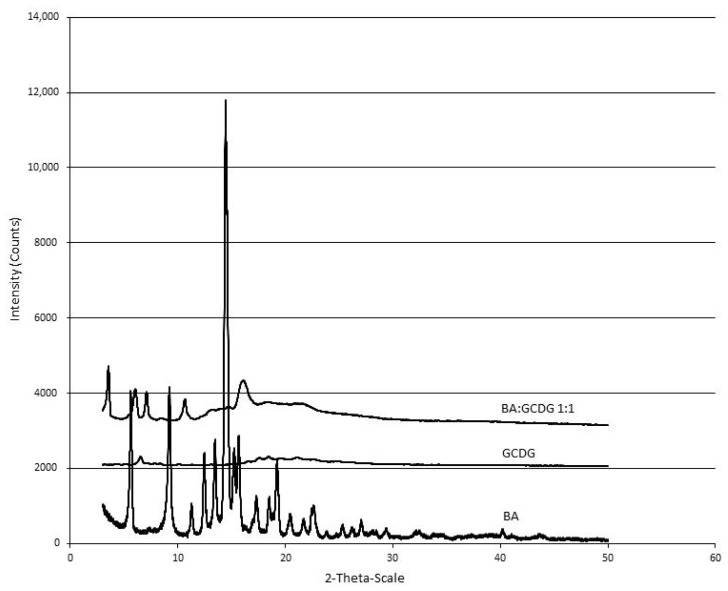
X-ray diffractograms of BA, GCDG and BA:GCDG 1:1. The measurements were conducted with a 50 kV tube voltage and tube current of 40 mA in step scan mode (step size 0.035, counting time 1 s per step).

X-ray analysis is typically used in the study of cyclodextrin complexing to characterize the changes that appear in the crystalline state of a compound as a result of intermolecular interaction. The X-ray diagrams of BA and GCDG are compared to the X-ray profile of the complex, revealing significant changes (Figure 4).

The BA X-ray diagram exhibits sharp peaks, which usually characterize crystalline compounds [15]. The cyclodextrin involved in the experiment reveals an almost flat X-ray diagram, with no distinguishable peaks, a profile that characterizes its amorphous nature. The diffractogram of the physical mixture of BA and GCDG (not shown) practically represents the sum of the individual diffractograms, revealing the BA peaks at a lower intensity compared with the pure compound in direct proportionality with its concentration in the mixture. The 1:1 complex provided a completely different X-ray diffractogram, in which several new peaks indicate the presence of a crystalline phase. Interestingly, the position of the new peaks differs substantially compared to the pure substance or its physical mixture with the cyclodextrin. The appearance of new peaks of different intensity and position can be considered evidence of real inclusion complex formation. Given the inclusion of BA inside of the GCDG cavity, BA-related peaks have disappeared, and a new compound emerged, characterized by a completely new X-ray profile.

### 2.4. MTT Assay

**Figure 5.**
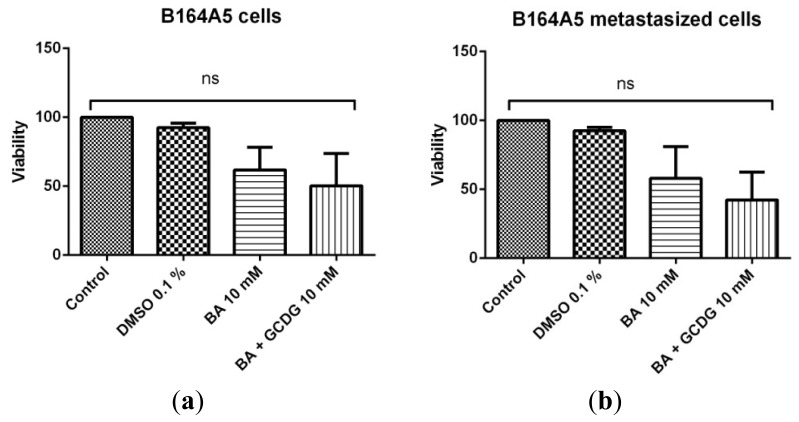
MTT assay showing the percentage of viable cells after 72 h of incubation with 10 mM BA and BA:GCDG 1:1 for non-metastatic (**a**) and metastatic (**b**) B164A5 cells. ns means not significant statistically.

The MTT proliferation assay indicated that BA inhibited the growth of both non-metastatic and metastatic B164A5 cells (Figure 5a,b). A slightly increased inhibitory activity on metastatic cells compared to the non-metastatic murine cell line was observed (57.89% viable cells *vs.* 61.82% viable cells, after 72 h of incubation). BA complexing with the newly synthesized cyclodextrin GCDG led to an improved antiproliferative activity, but the increase was not statistically relevant. After 72 h of incubation with the BA:GCDG complex, 50.30% of the B164A5 cells were viable, whereas only 42.33% of the metastatic B164A5 cells were viable under the same conditions. GCDG alone did not produce any significant effect on the non-metastatic or metastatic B164A5 cell proliferation. Our research group has previously reported on the antiproliferative effect of another triterpene with similar structure, betulin, on A431 (skin epidermoid carcinoma), A2780 (ovarian carcinoma), HeLa (cervical adenocarcinoma) and MCF7 (breast adenocarcinoma) [16] cell lines. Additionally, betulin, which differs from BA by a single functional group, was also described for its property to induce an antiproliferative effect on other human cell lines, such as hepatoma (HepG2) cells and lung adenocarcinoma (A549) cells [17]. Anticancer effects were also reported for neuroblastoma (SK-N-AS) cells and colon carcinoma (HT-29) cells [18]. However, as reported so far in several significant papers, BA remains the triterpene of choice for anticancer activity on both human and murine melanoma models. [1,19,20]. The conversion of betulin to BA follows a rather easy oxidative process [21]. The above mentioned results led us to the conclusion that in terms of antiproliferative effects, triterpene complexing with the newly synthesized CD is an interesting option to increase the antiproliferative activity. Increased anticancer activity for hydrophobic compounds after the incorporation in different types of CDs was previously reported in the literature [22–24]. Furthermore, by comparing the antiproliferative activity of BA on non-metastatic and metastatic B164A5 cells, we observed an increased sensitivity of the metastatic B164A5 cells compared to non-metastatic cells demonstrated by the percentage of viable cells, but the results were not statistically significant (*p* = 0.93).

### 2.5. Cell Cycle

The cell cycle analysis was conducted on both non-metastatic and metastatic B164A5 cells after incubation with BA and BA:GCDG 1:1 complex, respectively, and showed a G0/G1 arrest in both cases (Table 1). The capacity of BA to arrest the cell cycle in the G0/G1 phase was previously reported in the literature for various tumor cells lines, for example, Jurkat cells and cultured vascular smooth muscle cells [25–28]. BA cytotoxicity was noted to be selective on tumor cell lines but not on normal cells [4,29]. New semi-synthetic derivatives have been described to induce an S phase arrest for HepG2, HeLa and Jurkat cells [30]. By comparing the distribution in the phases of the cell cycle for non-metastatic and metastatic B164A5 cells after incubation with 10 mM BA for 72 h, we noticed that the percentage of cells blocked in the G0/G1 phase was increased in non-metastatic B164A5 cells as compared to the metastatic cells, having an average value of 15.55% cells blocked in the G0/G1 for non-metastatic B164A5 cells and 5.95% for metastatic B164A5 cells. Our results also showed that the incorporation of BA in GCDG increased the antiproliferative activity of the natural compound, leading to a higher number of cells blocked in the G0/G1 phase compared to pure BA. We observed that 23.29% of non-metastatic B164A5 cells and 15.41% of metastatic B164A5 cells were blocked in the G0/G1 phase. GCDG alone did not produce any significant effect on the cell cycle phases of the non-metastatic and metastatic B164A5 cells.

### 2.6. Annexin V-FITC-PI Double Staining Assay

**Figure 6.**
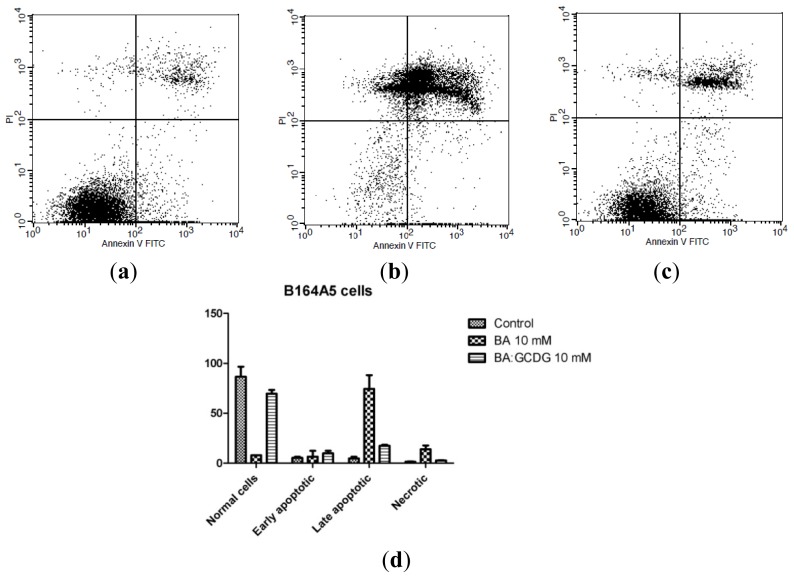
Representative dot-plots for Annexin V-FITC-PI-double staining after a period of incubation of 72 h for non-metastaticB164A5 cells: (**a**) Control; (**b**) BA 10 mM; (**c**) BA:GCDG 10 mM; and (**d**) quantification as percent viable cells during apoptosis (early and late apoptosis) and necrosis, using FACS software of the non-metastatic B164A5 cells: control, treated with BA dissolved in DMSO and BA:GCDG complex. Data are means ± SE of at least three independent experiments.

**Figure 7.**
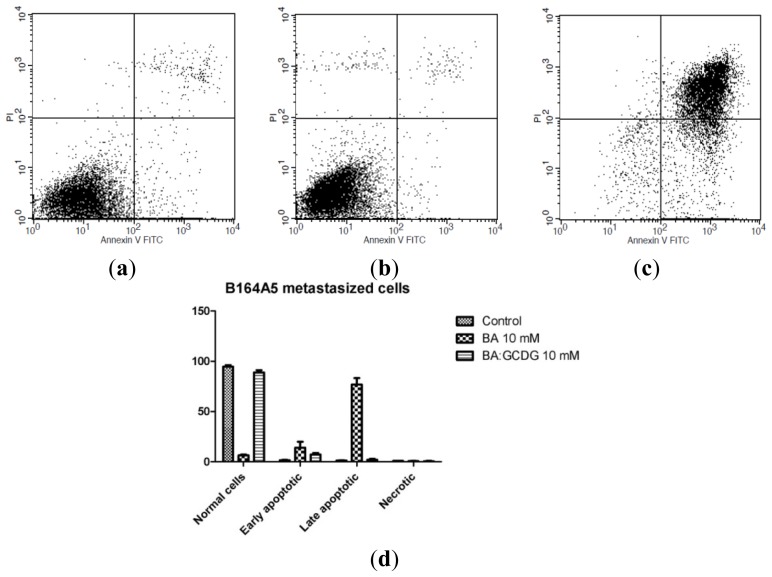
Representative dot-plots for Annexin V-FITC-PI-double staining after a period of incubation of 72 h for metastatic B164A5 cells: (**a**) Control; (**b**) BA 10 mM; (**c**) BA:GCDG 10 mM; and (**d**) quantification as percent viable cells during apoptosis (early and late apoptosis) and necrosis, using FACS software of the metastatic B164A5 cells: control, treated with BA dissolved in DMSO and BA:GCDG complex. Data are the mean ± SE of at least three independent experiments.

Annexin V-FITC-PI double staining is one of the most commonly employed assays to test the pro-apoptotic effects of active compounds [31]. BA has been previously reported to be a pro-apoptotic agent, causing late and early apoptosis on a wide range of cancer cell lines through the mitochondrial pathway [32,33]. Furthermore, BA cooperates with other anticancer drugs in inducing apoptosis [20]. Additionally, BA derivatives have been described for their pro-apoptotic activity on the B164A5 murine melanoma cell line [34]. The present study provides information concerning the activity of BA alone or incorporated in a hydrophilic CD, the newly synthesized GCDG, on non-metastatic and metastatic murine melanoma B164A5 cells. After 72 h of incubation of cells with 10 mM BA, late apoptosis was the predominant phenomenon detected. Early apoptosis, but not late apoptosis, was significantly different between the non-metastatic (5.08% early apoptotic cells; 74.47% late apoptotic cells) and metastatic cells (14.05% early apoptotic cells; 76.94% late apoptotic cells) (Figures 6 and 7). Under our experimental conditions, contrary to the beneficial effects recorded for the antiproliferative effect, it seems that in the context of apoptosis, BA incorporation in GCDG is an inappropriate approach due to a drastic decrease in the number of early and late apoptotic cells compared to pure BA. Based on the fact that similar results were previously reported in the literature for uremic media on endothelial cells and for interferon γ on murine astrocytes [35,36], we presume that the myc proto-oncogene family is involved in this behavior. Further investigation will be conducted to elucidate this outcome. GCDG alone did not produce any significant effect on the apoptosis of non-metastatic and metastatic B164A5 cells. The differences between the means were assessed using one way ANOVA followed by Bonferroni’s post-test, and *p* < 0.05 was considered statistically significant. Furthermore, to collect more data concerning the apoptotic events, we analyzed the expression of one initiator caspase, namely caspase 2. It is already known that the effector caspase 3 is stimulated after the incubation of B16 cells with BA [34]. Additionally, another pentacyclic triterpene, ursolic acid, was described to activate caspase 3 in another melanoma cell line, M4Beu [37]. Caspase 2 was not expressed by BA or BA:GCDG complex (Supplementary Information) treatment, suggesting that caspase 3 may be activated by another initiator caspase.

### 2.7. In Vivo Experiments

**Figure 8.**
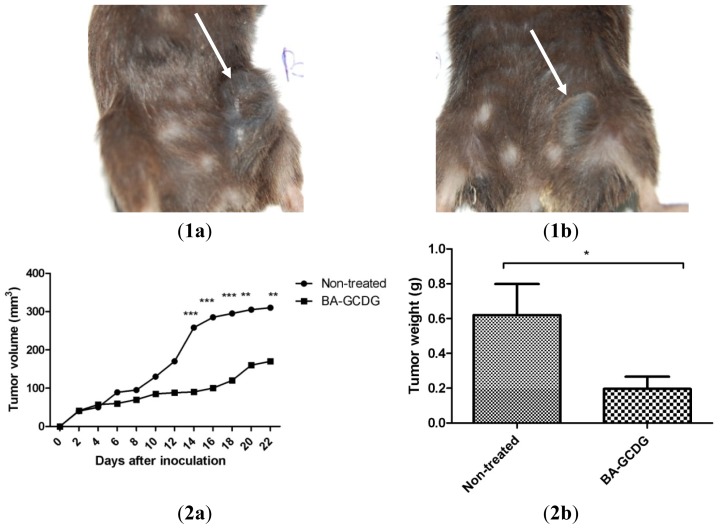
(**1**) Macroscopic images of tumor development on day 21 of the experiment developed in C57BL/6J mice: (**a**) Non-treated group (control group); and (**b**) BA:GCDG complex-treated group. (**2**) Tumor development: (**a**) Tumor volume (mm^3^); and (**b**) Tumor weight (g) for the treated and non-treated groups. Tumor growth (mm) was measured daily using a caliper, and tumor volume was estimated by using the following formula: length × width^2^/2. The administration of BA complex started on the first day after the murine melanoma cells were inoculated and was performed daily (100 mg/kg, intraperitoneally) until the final day of the experiment. Paired Student’s *t* tests or one-way ANOVA followed by Bonferroni’s post-tests were used; *p* < 0.05 (*), *p* < 0.01(**) and *p* < 0.001 (***) are indicated.

**Figure 9.**
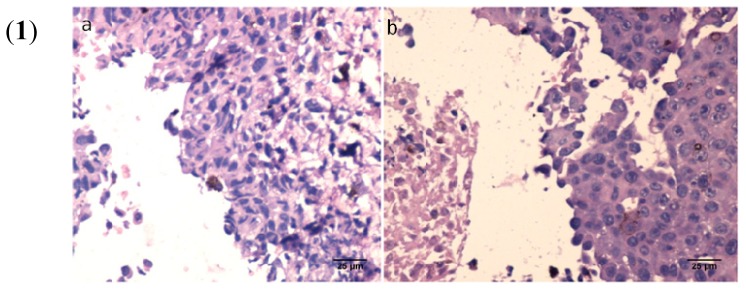

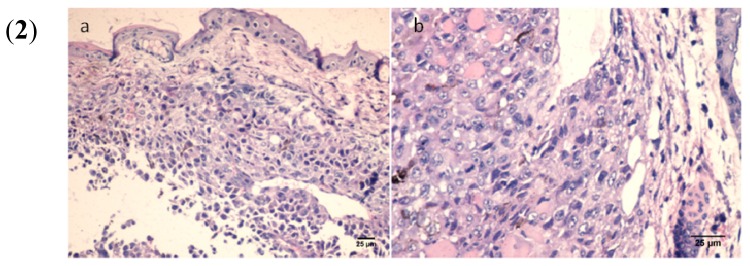
Histopathology of skin sections collected on day 21. (**1**) Group A (non-treated): (**a**) Tumor proliferation at the skin level characterized by the presence of elongated and oval cells with strong pleomorphism (HE ×400); and (**b**) Skin section with malignant melanoma detail with tumor necrosis (HE ×400). (**2**) BA complex-treated group: Tumor cells with vesicular nucleus, macronucleoli, atypical mitosis; (**a**) HE ×200; and (**b**) HE ×400.

**Figure 10.**
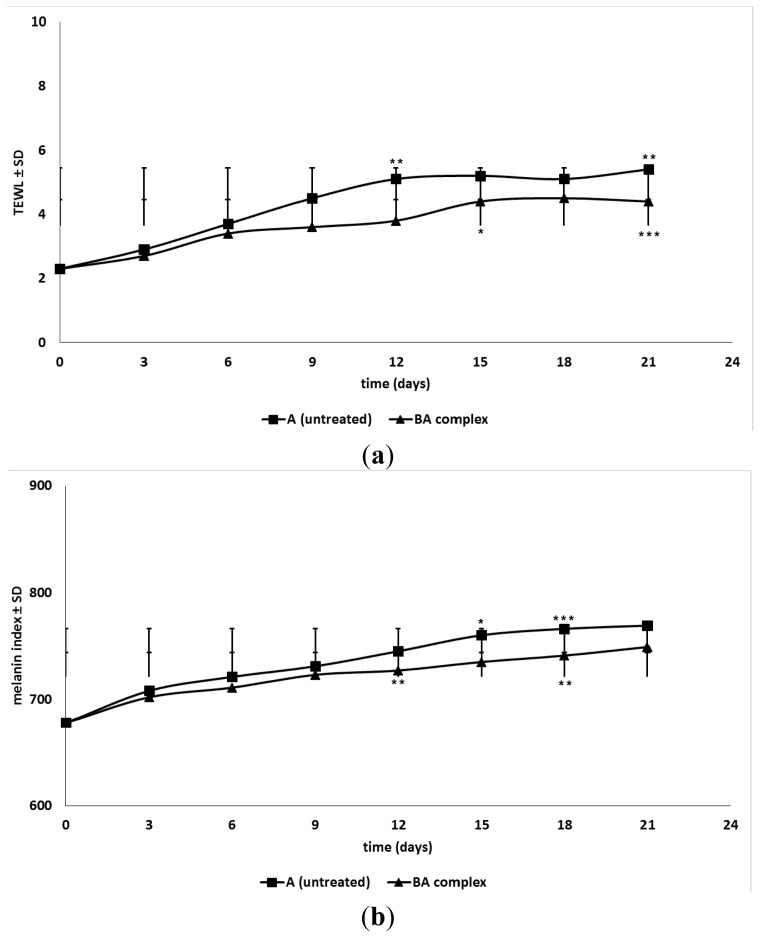

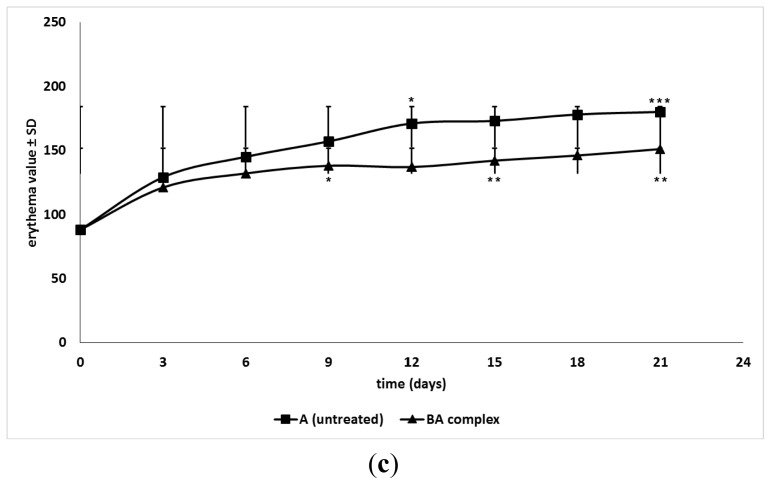
(**a**) Transepidermal water loss (TEWL); (**b**) Melanin index; and (**c**) Erythema evaluation on two experimental groups after 21 days of daily measurement. Statistic: Paired Student’s *t* tests or one-way ANOVA followed by Bonferroni’s post-tests were used; *p* < 0.05 (*), *p* < 0.01 (**) and *p* < 0.001 (***) are indicated.

To evaluate the effects of the BA:GCDG complex *in vivo*, we developed several experiments, including macroscopic follow-up (measurements of tumor volume/day and tumor weight after the mice were sacrificed—Figure 8, (1),(2)), histopathological analysis of tumor using the haematoxylin-eosin (HE) method (Figure 9, (1),(2)) and measurements of distinct skin parameters (transepidermal water loss (TEWL), melanin and erythema—Figure 10a–c).

Melanoma parameters in the context of tumor volume and weight were visibly reduced, as observed in the macro images (Figure 8, (1a),(1b)). Histological evaluation confirmed the differences between the two groups; for group A (non-treated), a more intense pathological process was observed, with a highly proliferative aspect (Figure 9, (1a),(1b)), while in group B, the presence of melanoma at the skin level was detected with lower semi-quantitative expression compared to the first group (Figure 9, (2a),(2b)).

Melanin and erythema values, recorded throughout the 21-day experiment (Figure 10b,c), revealed important changes in the pathological development of the two groups of animals. Group B (treated with BA:GCDG 1:1 complex) showed smaller values for melanin and erythema levels than group A (non-treated group). Regarding TEWL (Figure 10a), a significant parameter in skin evaluation, we noticed smaller values for the treated group (group B) compared to group A.

After the inoculation of murine cells into the colored mouse strain, one can notice the increase of melanin levels [38,39]; the B164A5 cell line is one of the colored murine melanoma cell types that leads to hyper pigmentation. Tumor development can be correlated with changes in the melanin index, as observed with smaller values in the treated group. Erythema values can be correlated, in most cases, with those of melanin [38]. The reduction of the skin hemoglobin level can be related to the pathology progression but also to an intervention in the local angiogenic process at the tumor site. BA has already been proven as an active antiangiogenic agent [40], data that are corroborated with the results of the present study. Moreover, a previous study [41] reported that BA induces differentiation (into corneocytes) and cell death in normal keratinocytes. Our study showed a significant influence of BA:GCDG treatment on the skin qualitative parameters, characterized by a decrease in TEWL, melanin and erythema (Figure 10a–c), indicating impaired tumor development, which can be translated as skin protection against tumor growth. These data may correlate with other studies using BA as a topical treatment that also provided skin protection [29,42].

BA is known as an active compound in the treatment of metastatic melanoma [43]. Additionally, it was successfully applied on experimental breast cancer and ovarian carcinoma xenografts [44,45]. In 2009, it was reported that BA suppresses tumor growth on several *in vivo* human xenograft models, including a melanoma model [29]. Murine melanoma also represents a target for the experimental application of BA. The murine B16/C57BL6 model rapidly metastasizes with a survival rate of approximately 30 days [42,46]. BA is characterized by a low water solubility that in many cases correlates with a low oral bioavailability [47] as well as the impossibility of a parenteral formulation. Increasing the solubility of several important antitumor compounds, such as paclitaxel [48], led to an improvement in tumor regression. This study investigated the possibility of the effect of BA in complex with a new highly soluble cyclodextrin derivative, GCDG. Substantial tumor regression was noticed in the animals of group B compared to the non-treated group A animals. The application of the water-soluble complexed BA was necessary to obtain a clear parenteral formulation; BA, being water insoluble, can only be administered orally. By increasing water solubility as a consequence of complexation, a better therapeutic outcome might likely be accomplished due to a superior distribution at the tumor level, as suggested by previous papers [49] and underscored by the decreased tumor volume observed in the treated group. BA administration in the earliest phase of the experiment was decided according to its important contribution in chemoprevention, as reported by Fulda in 2009 [29].

## 3. Experimental Section

GCDG was produced by organic synthesis at the Organic Macromolecular Chemistry Department, Saarland University, Saarbrucken, Germany and kindly given to us as a gift. Betulinic acid was purchased from Sigma Aldrich Ltd. (Taufkirchen, Germany, purity over 96%) and used as received. All other reagents were purchased and used as received.

### 3.1. Preparation of Complexes

Cyclodextrin complexes were prepared according to the literature using the kneading method. Briefly, the physical mixture of BA and GCDG was triturated with an equal quantity of ethanol and water (1:1 *v*/*v*). They were kneaded continuously for several minutes until most of the solvent had been evaporated; the resulted paste-type mixture was dried at room temperature (25 °C, normal atmospheric pressure) for 24 h and then at 105 °C for several hours in the oven until reaching constant weight. The final product was pulverized and sieved using a 100-μm sieve.

The binary complexes were prepared in a 1:1 BA:GCDG molar ratio.

### 3.2. Scanning Electron Microscopy

SEM pictures were captured using a JEM 100B electronic microscope (JEOL Ltd., Tokyo, Japan) functioning on scanning technique (JEM-ASID) with an accelerating voltage of 6 kV. Images were magnified by 200–10,000 times.

### 3.3. Differential Scanning Calorimetry

DSC measurements were carried out using a Mettler Toledo STAR Thermal Analysis System, DSC 821 (Mettler Inc., Schwerzenbach, Switzerland). The gas used as carrier was argon, the heating rate was maintained at 5 °C/min, while the sample weight was between 2–5 mg. Experiments were conducted from 25 up to 300 °C.

### 3.4. X-ray Diffraction

X-Ray-diffractograms were obtained using a Philips PW 1710 diffractometer, with Cu tube anode and a *K*α = 1.54242 Å. The measurements were conducted using a 50 kV tube voltage and 40 mA of tube current in step scan mode (step size 0.035, counting time 1 s per step).

### 3.5. Isolation of Metastatic Cells

Intraperitoneal metastases of approximately 5 mm^3^ were obtained from mice injected subcutaneously with melanoma tumor cells. Metastatic cells were isolated both using the explant and collagenase type IV-S from *Clostridim histolyticum* (Sigma Aldrich Company, Ayrshire, UK) methods. For the explant method, the metastatic samples were cut into small fragments and directly plated on cell culture-treated flasks. Tissue-isolated cells derived using the enzymatic digestion method were incubated for 15 min with collagenase at 37 °C, were passed through a series of phosphate buffer saline (PBS; Gibco BRL, Invitrogen, Carlsbad, CA, USA) washings, 0.70/0.40-μm strainer filtered and were replated as a single-cell suspension in adherent plastic culture plates. After isolation the cells were cultured and grown in Dulbecco’s Modified Eagle Medium (DMEM; Sigma Aldrich Company) supplemented with 10% fetal calf serum (FCS; PromoCell, Heidelberg, Germany) and 2% Penicillin/Streptomycin solution (PromoCell). The cells were kept at 37 °C in a humid atmosphere with 5% CO_2_. The culture medium was discarded every third day, and the cells were passaged when they reached 80%–90% confluence, using 0.25% Trypsin–EDTA (ethylenediaminetetraacetic acid) solution (Sigma Aldrich Company) followed by centrifugation (10 min, 300× *g*). After this step, the cells were seeded in T75 culture flasks at a density of 10,000 cells/cm^2^ to provide optimal growth and proliferation. Starting with the second passage, cells were partially used for further phenotypical analyses, while the subsequent experiments involved the metastatic cells expanded to passages 2–5.

All animal experiments complied with the European Convention for the Protection of Vertebrate Animals used for Experimental and Other Scientific Purposes (Directive 86/609, Strasbourg, 1986), and the experimental protocol was evaluated and approved by the “Victor Babeş” University of Medicine and Pharmacy, Timisoara, Ethics Committee and Board for Animal Experiments (IC 3/11.11.2009).

### 3.6. MTT Assay

B164A5 murine melanoma cells (5000 cells/well) (ECACC; Sigma Aldrich origin Japan stored UK) were seeded on a 96-well microplate. After 24 h, the test compounds were added in 200 μL of new medium containing Dulbecco’s Modified Eagle’s Medium (DMEM; Gibco BRL, Invitrogen, Carlsbad, CA, USA) supplemented with 10% fetal calf serum (FCS; PromoCell, Heidelberg, Germany) and 1% Penicillin/Streptomycin mixture (Pen/Strep, 10,000 IU/mL; PromoCell, Heidelberg, Germany) and incubated for 72 h.. Melanoma cells were treated with 5 mM EDTA and then passaged at confluence; the living cells were then evaluated by using 5 mg/mL MTT solution (20 μL). After 4 h incubation, MTT was precipitated as blue crystals by the mitochondrial reductase from the living cells. The medium was then discarded, and the blue MTT crystals were dissolved in 100 μL dimethyl sulfoxide (DMSO; Sigma-Aldrich Company, Ayrshire, UK). In the final step, the reduced MTT species were analyzed at 545 nm by a microplate reader; as control, the wells containing untreated cells were used. All *in vitro* experiments were simultaneously conducted on two microplates with at least five parallel wells. Stock solutions of the active substances were prepared using DMSO as the solvent (10 mM), and the highest final DMSO concentration (0.1%) of the medium did not show any significant impact on cell proliferation.

### 3.7. Cell Cycle

All B164A5 cells (control cells, BA, BA:GCDG and GCDG-treated cells, respectively) (10 mM active compound, 72 h incubation time) were submitted for cell cycle analysis performed using CycleTest™ Plus and a DNA Reagent Kit (Becton-Dickinson, San Jose, CA, USA). Briefly, this method comprises the following steps: dissolution of cell membrane lipids, elimination of the cellular cytoskeleton and nuclear proteins, RNA digestion, and the nuclear chromatin organization followed by propidium iodide (PI) binding to the clean, isolated nuclei. All procedures were carried out according to the manufacturer protocols and started with the interaction of the cells with 250 μL of Solution A (trypsin buffer) at room temperature for 10 min. In the second phase, 200 μL of Solution B (trypsin inhibitor and RNase buffer) was added, followed by a 10-min rest at room temperature; finally, 200 μL of cold (2–8 °C) Solution C (propidium iodide stain solution) was added, gently mixed and incubated for 10 min in the refrigerator protected from light.

Annexin V/PI assay data acquisition/cell cycle were performed on a four-color capable FACSCalibur (Becton-Dickinson) flow cytometer; data analysis was carried out using CellQuest Pro software (Becton-Dickinson).

### 3.8. Annexin V/PI Assay

All B164A5 cells (control cells, BA, BA:GCDG and GCDG-treated cells, respectively) (10 mM active compound, 72 h incubation time) were used in the apoptosis assay. Annexin V-FITC (Miltenyi Biotec, Gladbach, Germany) and Propidium Iodide Staining Solution (BD Biosciences, San Jose, CA, USA) were used in cell death flow cytometric analysis (apoptosis) according to the manufacturers’ protocols. Briefly, 10^6^ cells were washed in Annexin V Binding Buffer (BD Pharmigen, San Jose, CA, USA) and centrifuged at 300× *g*. Afterwards, the cells were resuspended using the same solution and incubated for 15 min with 10 μL of Annexin V-FITC, protected from light. The cells were washed with 1 mL of specific binding buffer and then centrifuged, and the resulted cell pellet was resuspended in 500 μL of binding buffer; 1 μg/mL PI solution was added, followed by flow cytometrical analysis.

### 3.9. Statistical Analysis

All experiments were carried out in triplicate to provide accurate statistical analysis. The results were statistically evaluated and expressed as the mean ± standard error. The differences between the means were analyzed using one way ANOVA followed by Bonferroni’s post-test; *p* < 0.05 was considered statistically significant.

### 3.10. Ethics Statement

All experimental procedures were evaluated and approved by the Ethical Committee of the Victor Babeş University of Medicine and Pharmacy, Timisoara, Romania. The experimental protocol was performed under the NIAH-National Institute of Animal Health rules; throughout the experiment, animals were kept under standard recommended conditions: 12 h light-dark cycle, *ad libitum* water and food, 24 °C temperature, and above 55% humidity). At the end of the experiment, the procedure for animals’sacrifice was cervical dislocation.

### 3.11. Animal Studies

Animal studies were conducted on 8-week-old C57BL/6J female mice (22–25 g average weight). The mice were purchased from Charles River (Sulzfeld, Germany). The experiment involved 20 animals equally divided into two groups as follows: group A, inoculated with B164A5 cells; group B, inoculated with B164A5 cells and then treated with BA complex in saline solution. We decided to use the group of mice that received no treatment as a control because of the results we obtained in one of our previous studies [46], where we analyzed the effect of GCDG both *in vitro* and *in vivo*, and no influence regarding a pharmacological effect or toxicity of this compound was detected.

B164A5 (ECACC and Sigma Aldrich, origin Japan stored UK) cells were processed as described in the MTT assay protocol. On day 0 of the experiment, the animals from the two groups, A and B, were subcutaneously inoculated, into the hair-depilated lateral abdomen, with 0.1 mL of 1 × 10^5^ cells/mouse [38]. The mice were monitored daily throughout the experiment so that the development of tumors or other significant changes could be noticed. Tumors became clear and visible on day 9 after inoculation. Tumor growth (mm) was measured daily using a caliper, and tumor volume was estimated using the following formula: length × width^2^/2 [50]. The administration of the BA complex to animals from group B started on the first day after the murine melanoma cell inoculation and was performed daily (100 mg/kg, intraperitoneally) until the final day of the experiment. Because BA is water-insoluble, it could not be administered as an aqueous solution. The experiment lasted 21 days from the inoculation; the survival rate was 100% for both groups. Tumors were collected, measured, weighed and analyzed by classical histological techniques. The results are expressed as the mean ± SD Statistical analysis were performed for tumor volume and weight using one-way ANOVA followed by Bonferroni’s post-test or paired Student’s *t* tests, depending on the case; *p* < 0.05 was considered significant.

### 3.12. Non-Invasive Skin Measurements

For all non-invasive measurements carried out on the mouse skin, a Multiprobe Adapter System (MPA5) from Courage-Khazaka (Cologne, Germany) was used; the melanin and erythema measurements were performed using a Mexameter^®^ MX 18 (Courage-Khazaka) that offers quantitative information regarding the melanin and erythema (hemoglobin) level subjected to modifications following tumor development with or without treatment. The device emits light over 3 wavelengths (568, 660 and 880 nm) and measures the remitted light over a 5-mm diameter. The erythema and melanin indices are determined as follows:

(1) $$Mx=\frac{500}{\text{log }5}\text{log}\frac{\text{Infrared}}{\text{Red}}+\text{log }5$$

(2) $$Ex=\frac{500}{\text{log }5}\text{log}\frac{\text{Red}}{\text{Green}}+\text{log }5$$

where *Mx* = melanin index; *Ex* = erythema index; and infrared/red/green = infrared/red/green remittance.

These indices are relative values, and the maximum ratio between each color is 1:5. The values are within the range of 0–1000, with a higher value representing an increased melanin or erythema level, while a value of 500 represents a remittance ratio of 1:1. All measurements were carried out in four skin regions located near the tumor, and mean and standard deviation were calculated [51]. Statistical tests were used to determine the significant differences between the two experimental groups. The amount of melanin and erythema was measured at baseline (day 0) and then every other day until day 21 of the experiment. The measurement area was 5 mm in diameter. At the end of experiment, mice were sacrificed, and the skin was collected and histologically analyzed.

### 3.13. Histology

For the histological analysis, tissue samples (skin) were fixed in a 10% formalin solution and then embedded in paraffin and cut at four microns. Finally, after the removal of paraffin, the samples were stained with hematoxylin-eosin (HE) and microscopically analyzed.

## 4. Conclusions

BA is an active agent for the treatment of murine metastatic melanoma. It has a significant negative impact on tumor growth, leading to a reduced pathology and an increased survival time. Moreover, BA is a skin protective agent and, consequently, an important candidate for *in vivo* melanoma treatment.

## Supplementary Information



## Figures and Tables

**Table 1.** Evaluation of cell cycle phases variation between control, B164A5 and B164A5 metastatic melanoma cells treated with GCDG, BA and their 1:1 complex.

Sample (10 mM)	Cell cycle phases for non-metastatic B164A5 cells				Cell cycle phases for metastatic B164A5 cells			
	
	under-G0 (%)	G0/G1 (%)	S (%)	G2/M (%)	under-G0 (%)	G0/G1 (%)	S (%)	G2/M (%)
Control	2.88 ± 0.14	64.32 ± 2.13	21.17 ± 0.22	11.63 ± 0.17	1.24 ± 1.28	71.68 ± 3.14	18.43 ± 1.19	8.65 ± 1.21
DMSO	1.49 ± 0.22	69.43 ± 1.39	19.81 ± 0.34	9.27 ± 0.31	1.93 ± 1.17	68.39 ± 2.67	17.03 ± 0.28	12.65 ± 1.19
BA	0.79 ± 3.4	87.61 ± 1.64	6.14 ± 0.28	5.46 ± 0.25	1.39 ± 2.21	87.09 ± 3.03	7.45 ± 1.65	4.07 ± 3.42
BA:GCDG	2.29 ± 1.27	79.87 ± 1.94	11.98 ± 1.73	5.86 ± 2.01	2.97 ± 2.27	77.53 ± 2.29	8.29 ± 3.02	11.21 ± 1.98
