# Supplementary material for: Betulinic Acid in Complex with a Gamma-Cyclodextrin Derivative Decreases Proliferation and in Vivo Tumor Development of Non-Metastatic and Metastatic B164A5 Cells
Source: Int J Mol Sci. 2014 May 9;15(5):8235–55. doi: 10.3390/ijms15058235 (PMC4057729; doi:10.3390/ijms15058235)
Supplement: Supplementary file 1 [file ijms-15-08235-s001.pdf]

Supplementary Information

Caspase 2 Detection

Immunocytochemistry was performed for normal and metastasized B16 4A5 cells at a density of 10,000 cells/cm^2 in 4-well glass chamber slides (Nalgene Nunc International, New York, NY, USA) and expanded for 24 h in culture medium. After 24 h, new medium containing 10 mM BA and 10 mM BA:GCDG, respectively, were added. Control well contained untreated cells. Cells were maintained in culture for 72 h after addition of substances, and then prepared for immunocytochemical staining. After removing the culture medium, cells were washed, fixed with 4% paraformaldehyde (Sigma-Aldrich Company, Taufkirchen, Germany), permeabilized with 0.1% Triton X-100 (Sigma-Aldrich Company) and then investigated for the expression of the proteins of interest, using for labeling anti-h/m Caspase 2 (mCaspase 2 affinity purified rabbit IgG) (R&D Systems, Abingdon, UK). Staining protocol continued with secondary biotinylated antibody binding and substrate addition (AEC) (Dako EnVision™ + System-HRP, Dako, Carpinteria, CA, USA) following the manufacturer's protocol. After counterstaining with hematoxylin solution (Hematoxylin, Mayer's Lillie's Modification, Dako) for 30 s and washing with tap water the slides were mounted in an aqueous mounting media (Crystal/Mount™, Biomeda, CA, USA). Microscopy analysis was performed on a Nikon Eclipse E800 microscope (Tokyo, Japan).

Figure S1. Caspase 2 staining after a period of incubation of 72 h for normal B164A5 cells: (**a**) Control; (**b**) BA 10 mM; (**c**) BA:GCDG 10 mM; and (**d**) GCDG 10 mM.

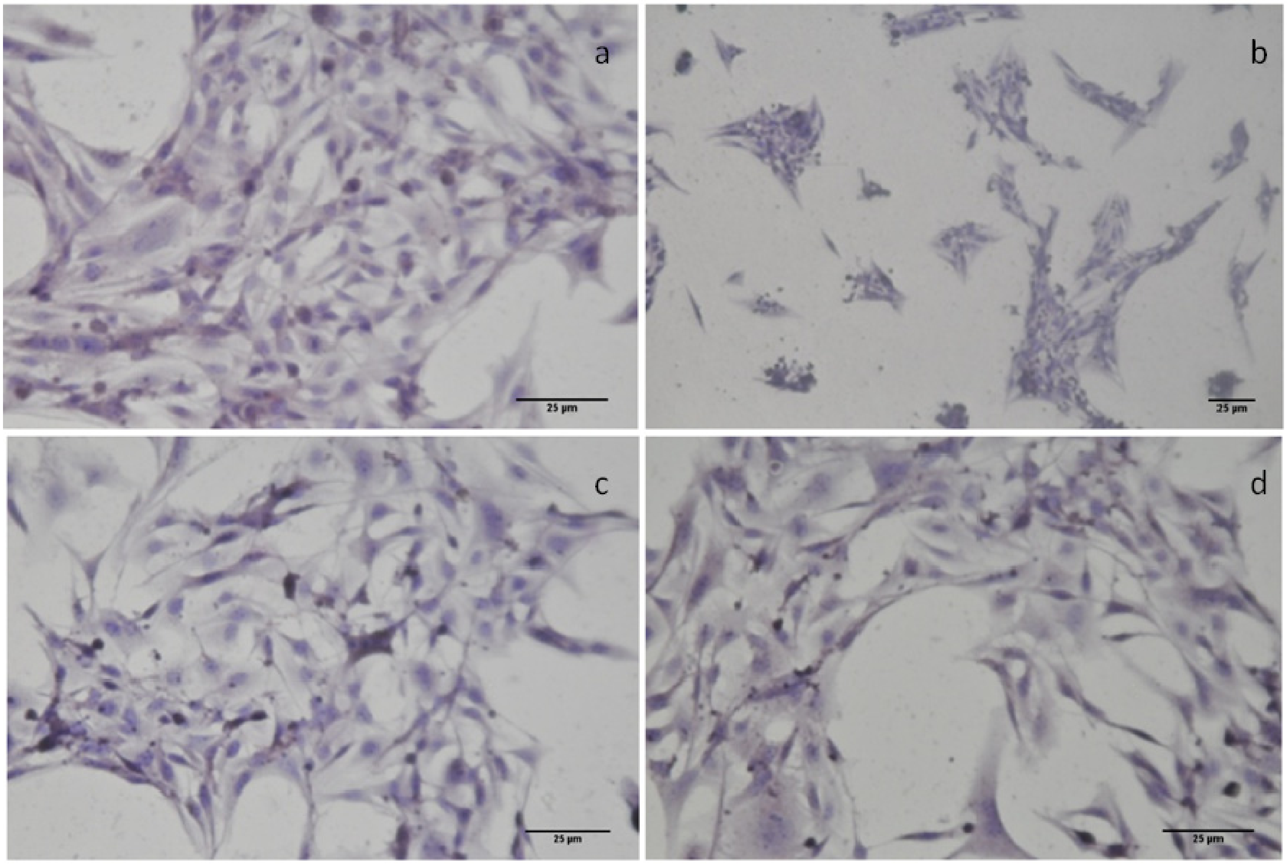

Figure S2. Caspase 2 staining after a period of incubation of 72 h for metastatic B164A5 cells: (**a**) Control; (**b**) BA 10 mM; (**c**) BA:GCDG 10 mM; and (**d**) GCDG 10 mM.

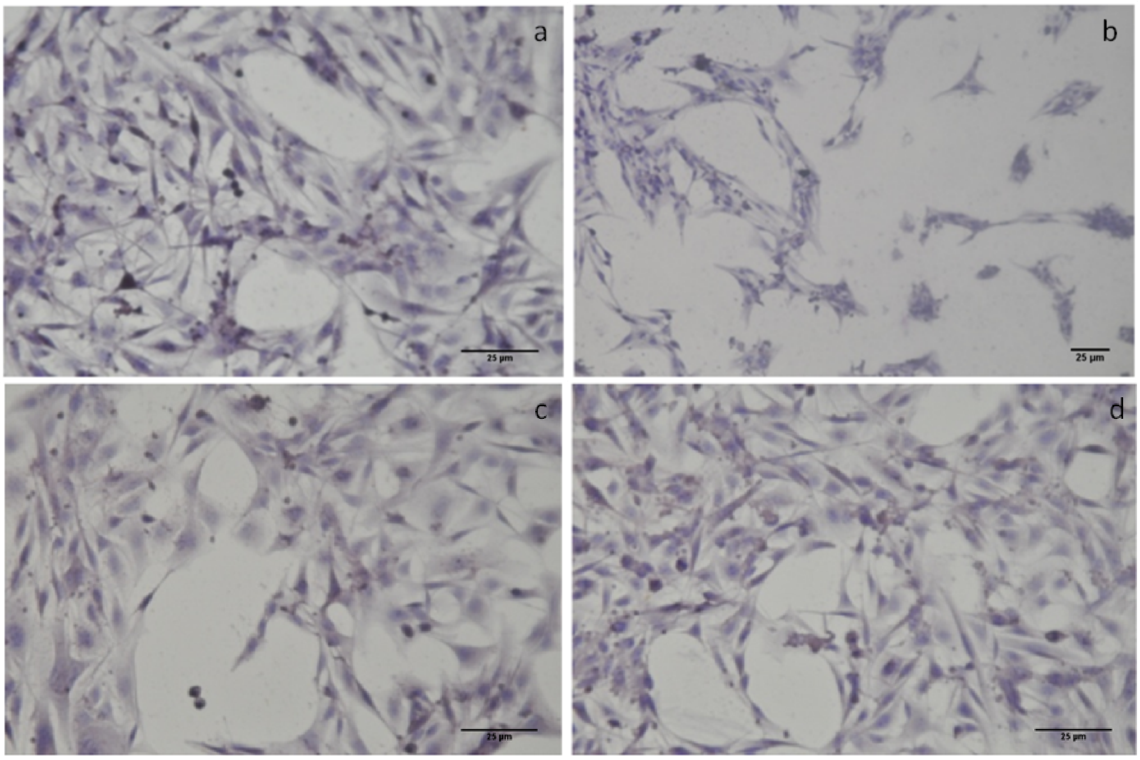

© 2014 by the authors; licensee MDPI, Basel, Switzerland. This article is an open access article distributed under the terms and conditions of the Creative Commons Attribution license (http://creativecommons.org/licenses/by/3.0/).
